# THREE-MONTH OUTCOMES OF ISCHEMIC COLITIS IN ANOREXIC VS NON-ANOREXIC PATIENTS: A PROPENSITY-MATCHED COHORT STUDY

**DOI:** 10.1590/S0004-2803.24612025-102

**Published:** 2026-05-18

**Authors:** Chidera ONWUZO, FNU ALVINA, Rashid ABDEL-RAZEQ, Kofi AWUAH, Somtochukwu ONWUZO

**Affiliations:** 1 SUNY Upstate Medical University, Department of Medicine, Syracuse, NY, United States.; 2 Cleveland Clinic Foundation, Department of Internal Medicine, Cleveland, OH, United States.; 3 Allegheny Health Network, Pittsburgh, PA, United States.

**Keywords:** Anorexia nervosa, ischemic colitis, colectomy, parenteral nutrition, surgical complications, Anorexia nervosa, colite isquêmica, colectomia, nutrição parenteral, complicações cirúrgicas

## Abstract

**Background:**

Ischemic colitis arises from a reduction in mesenteric blood flow, a condition often aggravated by factors such as severe dieting observed in individuals with anorexia nervosa which compromise the gastrointestinal vasculature, increasing the susceptibility to ischemic injury. **Objective:** This study aims to rigorously compare three-month clinical outcomes, including colectomy rates, mortality, and other complications, between anorexic and non-anorexic patients diagnosed with ischemic colitis.

**Methods:**

A validated multicenter database of more than 70 different healthcare systems across the United States was utilized. The population was divided into, Cohort 1 that consisted of anorexic patients with ischemic colitis and Cohort 2 which consisted of non-anorexic patients with ischemic colitis. After propensity score matching, a total of 450 patients were identified in each cohort. Outcomes were assessed over a 90-day period following the index event of ischemic colitis diagnosis. Kaplan-Meier curves and risk analyses were conducted to evaluate mortality, parenteral nutrition requirement, PEG placement and colectomy rates.

**Results:**

At three months, anorexic patients demonstrated significantly higher colectomy rates odds ratio (OR) of 2.262 (95%CI: 1.269-4.031, *P*=0.005) and mortality with an OR of 1.462 (95%CI: 1.079-1.981, *P*=0.014) compared to their non-anorexic counterparts. The differences in PEG insertion and PN (parenteral nutrition) use were not statistically significant.

**Conclusion:**

Anorexic patients with ischemic colitis experience significantly worse short-term outcomes, including higher rates of mortality and colectomy, compared to their non-anorexic counterparts.

## HIGHLIGHTS

• Propensity-matched U.S. multicenter cohort (450 per group).

• Higher 90-day colectomy in anorexic patients (OR 2.26; *P*=0.005).

• Higher 90-day mortality in anorexic patients (OR 1.46; *P*=0.014).

• No significant difference in PEG or parenteral nutrition.

• Anorexia is linked to worse short-term ischemic colitis outcomes.

## RESUMO

**Contexto:**

A colite isquêmica resulta da redução do fluxo sanguíneo mesentérico, uma condição frequentemente agravada por fatores como dietas restritivas observadas em indivíduos com anorexia nervosa, que comprometem a vascularização gastrointestinal, aumentando a suscetibilidade a lesões isquêmicas.

**Objetivo:**

Este estudo visa comparar rigorosamente os desfechos clínicos em três meses, incluindo taxas de colectomia, mortalidade e outras complicações, entre pacientes anoréxicos e não anoréxicos diagnosticados com colite isquêmica.

**Métodos:**

Foi utilizada uma base de dados multicêntrica validada, abrangendo mais de 70 sistemas de saúde diferentes nos Estados Unidos. A população foi dividida em duas coortes: a Coorte 1, composta por pacientes anoréxicos com colite isquêmica, e a Coorte 2, composta por pacientes não anoréxicos com colite isquêmica. Após o pareamento por escore de propensão, foram identificados 450 pacientes em cada coorte. Os desfechos foram avaliados durante um período de 90 dias após o diagnóstico inicial de colite isquêmica. Curvas de Kaplan-Meier e análises de risco foram realizadas para avaliar a mortalidade, a necessidade de nutrição parenteral, a colocação de sonda de gastrostomia endoscópica percutânea (PEG) e as taxas de colectomia.

**Resultados:**

Aos três meses, pacientes anoréxicos apresentaram taxas significativamente maiores de colectomia, com uma razão de chances (OR) de 2,262 (IC95%: 1,269-4,031, *P*=0,005) e de mortalidade, com uma OR de 1,462 (IC95%: 1,079-1,981, *P*=0,014), em comparação com pacientes não anoréxicos. As diferenças na inserção de sonda de gastrostomia endoscópica percutânea (PEG) e no uso de nutrição parenteral (NP) não foram estatisticamente significativas.

**Conclusão:**

Pacientes anoréxicos com colite isquêmica apresentam desfechos de curto prazo significativamente piores, incluindo maiores taxas de mortalidade e colectomia, em comparação com pacientes não anoréxicos.

## INTRODUCTION

Anorexia nervosa (AN) is a rare but potentially life-threatening psychiatric condition with multiple complications ranging from severe weight loss, malnutrition and severe multiorgan damage. It predominantly affects adolescent women^1^ ^-^ ^4^. According to the American Psychiatric Association’s Diagnostic and Statistical Manual (DSM-V), all 3 of the following criteria must be met for a diagnosis of anorexia nervosa: A) restriction of energy intake relative to requirements, leading to a significant low body weight in the context of the age, sex, developmental trajectory, and physical health. B) intense fear of gaining weight or becoming fat or persistent behavior that interferes with weight gain. C) disturbance in the way body weight is perceived^5^.

Ischemic colitis is characterized by an acute reduction in blood flow to the colon, which can lead to varying degrees of intestinal necrosis and damage. The most common risk factors predisposing to this condition include age greater than 60 years, diabetes mellitus, hypertension, dyslipidemia, peripheral vascular disease, cerebral infarction, hypoalbuminemia, and constipation^6^ ^,^ ^7^ Mesenteric venous thrombosis can also cause intestinal ischemia and is usually related to thrombophilia, trauma, or local inflammatory changes such as pancreatitis and diverticulitis^8^ ^-^ ^10^.

There are various pathophysiological mechanisms leading to gastrointestinal dysfunction in patients with Anorexia Nervosa. These include delays in gastric motility, gastric emptying and intestinal transit. They may also occur due to severe malnutrition leading to electrolyte imbalances or from symptoms such as self-induced purging or even the refeeding process. They contribute to various complications, by impairing both the structure and functional integrity of the gastrointestinal tract leading to decreased blood flow^11^ ^-^ ^13^.

Ischemic colitis is a very rare complication of AN, there are very few reported cases of anorexia nervosa resulting in small bowel and colonic ischemia, the majority resulting in mortality^14^ ^-^ ^17^.

This study aims to rigorously compare three-month clinical outcomes, including colectomy rates, mortality, and other complications, between anorexic and non-anorexic patients diagnosed with ischemic colitis, providing valuable insight into the unique risks and management challenges faced by these patients.

## METHODS

**FIGURE 1:**
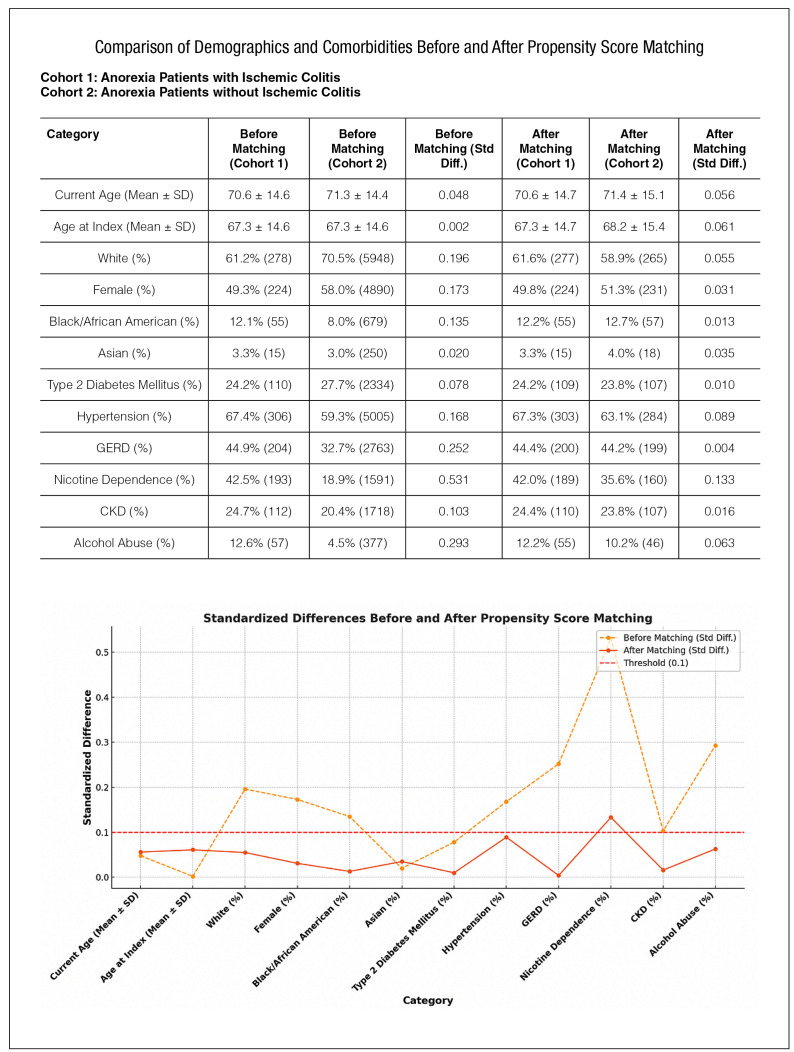
Comparison of demographics and comorbidities of anorexic group vs non anorexic group before and after propensity score matching.

A validated multicenter and research platform database of more than 70 different healthcare systems with over 130 million patients across the United States was utilized to construct this study. Patients aged ≤18 years were excluded. Studied patient population was divided into, Cohort 1 that consisted of anorexic patients with ischemic colitis and Cohort 2 which consisted of non-anorexic patients with ischemic colitis. After propensity score matching, a total of 450 patients were identified in each cohort (Figure 1). Outcomes were assessed over a 90-day period following the index event of ischemic colitis diagnosis. Kaplan-Meier curves and risk analyses were conducted to evaluate mortality, parenteral nutrition requirement, percutaneous endoscopic gastrostomy (PEG) tube placement and colectomy rates.

## RESULTS

**FIGURE 2:**
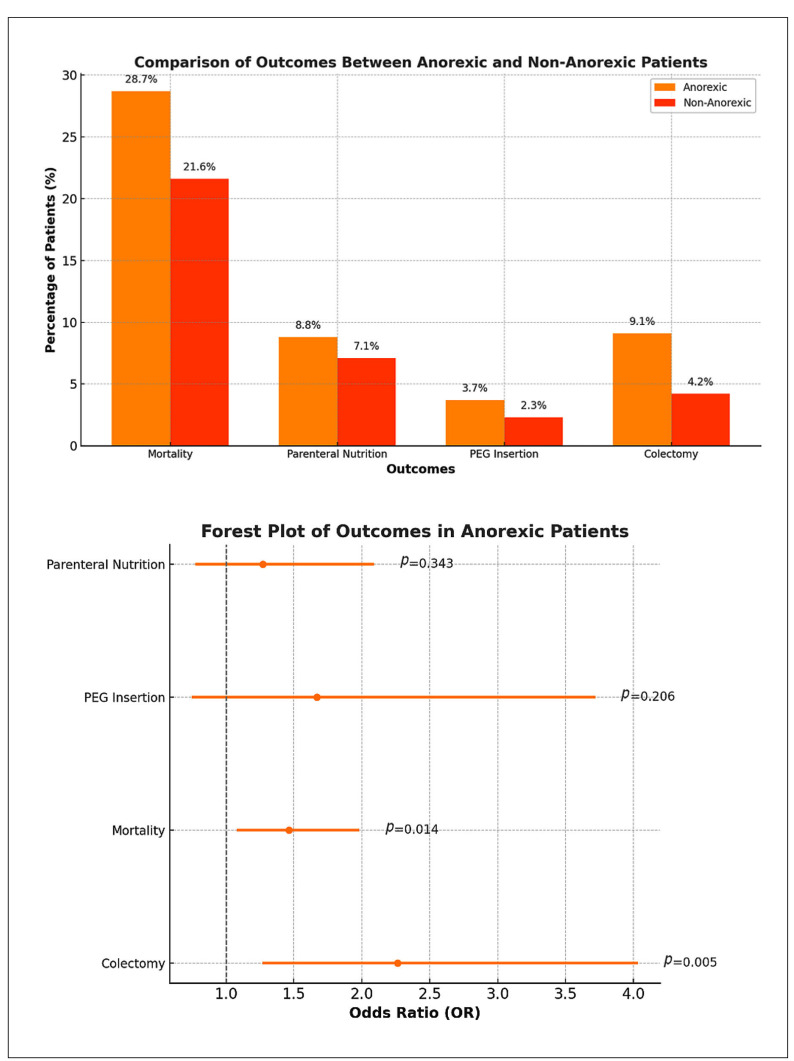
Rates of outcomes and forest plot depicting odds of events occuring between anoxeric and non anoxeric groups.

At three months, anorexic patients demonstrated significantly higher colectomy rates compared to their non-anorexic counterparts, with an odds ratio (OR) of 2.262 (95%CI: 1.269-4.031, *P*=0.005), depicting a strong association between anorexia and the increased need for surgical intervention. Mortality was also markedly elevated in anorexic patients, with an OR of 1.462 (95%CI: 1.079-1.981, *P*=0.014), reflecting a heightened risk of death in this population. PEG insertion was more frequent among anorexic patients (3.7%) compared to non-anorexic patients (2.3%), this difference was not statistically significant (OR 1.669, 95%CI: 0.749-3.720, *P*=0.206). Similarly, the use of parenteral nutrition was higher in anorexic patients (8.8%) as compared to non-anorexic patients (7.1%), however there was no statistically significant association, with an OR of 1.272 (95%CI: 0.773-2.091, *P*=0.343) (Figure 2). These findings highlight the critical and complex nature of managing anorexia-associated ischemic colitis, with a pronounced impact on surgical and survival outcomes.

## DISCUSSION

The primary outcome in our study demonstrated significantly worse 3-month outcomes of colectomy and mortality in anorexic patients with ischemic colitis compared to non-anorexic patients. The anorexic cohort demonstrated significantly higher colectomy rates (OR 2.262, *P*=0.005) as compared to the non-anorexic patient cohort.

Mortality was also markedly elevated in anorexic patients, with an OR of 1.462 (95%CI: 1.079-1.981, *P*=0.014), reflecting a heightened risk of death in this population and the increased need for surgical intervention in the anorexic population. The pathophysiology results from non-occlusive mesenteric ischemia, leading to necrotizing ischemic colitis caused due to dehydration, severe malnutrition and electrolyte imbalances leading to decreased perfusion in the mesenteric circulation^18^ ^,^ ^19^.

Sakka S et al., in their early case report describe a fulminant case of necrotizing colitis in a young female patient with anorexia nervosa requiring colectomy and emphasize on ischemic colitis as a true complication of anorexia nervosa^17^. In their case report and literature review, Strand et al. emphasize the necessity of urgent surgical intervention for ischemic colitis in patients with anorexia nervosa-highlighting its high mortality rate of up to 80%-and note that all survivors underwent either partial or complete bowel resection^20^.

Foran AT et al., describe another case report of a young female with anorexia nervosa who developed ischemic colitis and underwent a hemi-colectomy. The report further highlights the frequency of colectomy rates and the importance of appropriate radiological and surgical input^21^.

PEG insertion was more prevalent among anorexic patients, but there was no statistically significant difference (OR 1.669, 95%CI: 0.749-3.720, *P*=0.206). Similarly, the use of parenteral nutrition (PN) was more common in anorexic patients but showed no statistically significant association with anorexia, with an OR of 1.272 (95%CI: 0.773-2.091, *P*=0.343). Schapira B et al. is their case report describe a female patient with necrotizing colitis requiring bowel resection managed with parenteral nutrition and bowel rest for 7 days post operatively following which enteral nutrition was reintroduced^19^.

Diamanti A et al. emphasize the critical role that nutrition plays in the recovery of malnourished anorexic patients with ischemic colitis and describes parenteral nutrition as the cornerstone to aid recovery when enteral nutrition is not tolerated. The report emphasizes the better survival outcomes associated with nutritional supplementation^15^. Enteral feeding via PEG is recommended in anorexic patients who require chronic supplemental nutrition due to the risks associated with long-term TPN^15^ ^,^ ^22^.

The mean age of patients in both cohorts (approximately 70-71 years) aligns with the understanding that ischemic colitis is most common in older adults^7^.

Hypertension, diabetes mellitus, and cerebral infarction have been identified as the most common risk factors and comorbidities for ischemic colitis in population-based studies^23^.

Cubiella Fernández J. et al., in their case-control study identified that diabetes, dyslipidemia, heart failure, peripheral arterial disease and use of digoxin and aspirin were independently associated with the development of ischemic colitis^7^.

The higher rate of alcohol abuse in anorexic patients with ischemic colitis is consistent with previous findings. Watahiki Y. et al., in their study identified alcohol consumption as potentially increasing the risk of mesenteric ischemia through multiple mechanisms, including suppression of fibrinolysis and mesenteric vasoconstriction by suppressing endothelium-dependent vasorelaxation^24^.

The propensity score matching balanced most variables, reducing them below the threshold of 0.1. Nicotine dependence was slightly above the threshold (std diff=0.133). This confirms that anorexia is independently associated with increased rates of colectomy and mortality independent of demographic characteristics and comorbid conditions.

The high prevalence of nicotine dependence among anorexic patients with ischemic colitis aligns with established research. Sherid M. et al., in their multicenter retrospective study found that current smoking status was identified as a significant risk factor for recurrence of ischemic colitis, with 50% of patients in the recurrent ischemic colitis group being current smokers compared to only 18.7% in the non-recurrent group^25^. Karban A et al., in their study indicate that smoking affects intestinal blood flow differently depending on the site of inflammation, potentially explaining the differing effects on various gastrointestinal conditions^26^.

The limitations of this study include the potential for bias due to unmeasured confounders such as the nutritional status of patients beyond anorexia, socio-economic status, treatment adherence and survivor bias. Although the data was obtained from a validated multicenter and research platform database of more than 70 different healthcare systems with over 130 million patients across the United States, the electronic records might have errors in timely data entry of lab values, comorbidities and even diagnosis. The propensity matching model often discards a wide population of unmatched cases, thus reducing the power of the unmatched pool. This could be the reasoning behind the more uncommon conditions like PEG insertion and TPN not reaching statistical significance.

Although there are multiple case reports documenting ischemic colitis as a complication of anorexia nervosa^14^ ^-^ ^17^, there is a paucity of studies describing the clinical outcomes and mortality rates in this population. This highlights the importance for increased clinical vigilance, prevention strategies, and the need for early intervention protocols for ischemic colitis in patients with anorexia nervosa.

Despite the findings of our retrospective study demonstrating a significant association between anorexia nervosa and increased rates of colectomy and mortality, prospective controlled trials are needed to address hidden confounding factors and form a causal relationship.

## CONCLUSION

Anorexic patients with ischemic colitis experience significantly worse short-term outcomes, including higher rates of mortality and colectomy, compared to their non-anorexic counterparts. These findings reflect the importance of prompt recognition and aggressive management of ischemic colitis in this vulnerable population, where preexisting physiological stressors such as severe malnutrition and compromised vascular integrity may exacerbate disease severity.

## Data Availability

Data in article: the research data are presented within the article itself (available in Figure 1 and Figure 2).
